# A pilot study on the kinetics of metabolites and microvascular cutaneous effects of nitric oxide inhalation in healthy volunteers

**DOI:** 10.1371/journal.pone.0221777

**Published:** 2019-08-30

**Authors:** Adriano R. Tonelli, Kulwant S. Aulak, Mostafa K. Ahmed, Alfred Hausladen, Batool Abuhalimeh, Charlie J. Casa, Stephen C. Rogers, David Timm, Allan Doctor, Benjamin Gaston, Raed A. Dweik

**Affiliations:** 1 Department of Pulmonary, Allergy and Critical Care Medicine, Respiratory Institute, Cleveland Clinic, Cleveland, OH, United States of America; 2 Pathobiology Division, Lerner Research Institute, Cleveland Clinic, OH, United States of America; 3 Inflammation and Immunity, Lerner Research Institute, Cleveland Clinic, OH, United States of America; 4 Department of Chest Diseases, Faculty of Medicine, Assiut University, Assiut, Egypt; 5 Institute for Transformative Molecular Medicine and Department of Medicine, Case Western Reserve University School of Medicine and University Hospitals Cleveland Medical Center, Cleveland, OH, United States of America; 6 Department of Pediatrics, University of Maryland School of Medicine, Baltimore, MD, United States of America; 7 Department of Psychiatry, School of Medicine, Washington University, St. Louis, MO, United States of America; 8 Herman B Wells Center for Pediatric Research, Indiana University School of Medicine, Indianapolis, IN, United States of America; Bauer Research Foundation, UNITED STATES

## Abstract

**Rationale:**

Inhaled nitric oxide (NO) exerts a variety of effects through metabolites and these play an important role in regulation of hemodynamics in the body. A detailed investigation into the generation of these metabolites has been overlooked.

**Objectives:**

We investigated the kinetics of nitrite and S-nitrosothiol-hemoglobin (SNO-Hb) in plasma derived from inhaled NO subjects and how this modifies the cutaneous microvascular response.

**Findings:**

We enrolled 15 healthy volunteers. Plasma nitrite levels at baseline and during NO inhalation (15 minutes at 40 ppm) were 102 (86–118) and 114 (87–129) nM, respectively. The nitrite peak occurred at 5 minutes of discontinuing NO (131 (104–170) nM). Plasma nitrate levels were not significantly different during the study. SNO-Hb molar ratio levels at baseline and during NO inhalation were 4.7E-3 (2.5E-3–5.8E-3) and 7.8E-3 (4.1E-3-13.0E-3), respectively. Levels of SNO-Hb continued to climb up to the last study time point (30 min: 10.6E-3 (5.3E-3-15.5E-3)). The response to acetylcholine iontophoresis both before and during NO inhalation was inversely associated with the SNO-Hb level (r: -0.57, p = 0.03, and r: -0.54, p = 0.04, respectively).

**Conclusions:**

Both nitrite and SNO-Hb increase during NO inhalation. Nitrite increases first, followed by a more sustained increase in Hb-SNO. Nitrite and Hb-SNO could be a mobile reservoir of NO with potential implications on the systemic microvasculature.

## Introduction

Over the past years the role of NO in biology has increased tremendously and is involved in diverse functions such as bacterial defense, neurotransmission and reproduction [1–4]. NO is a highly diffusible gas synthesized by a group of nitric oxide synthases (NOS) [5]. It was identified in 1980s and initially referred as endothelium-dependent relaxation factor [6–8]. Three isoforms of NOS exist but the endothelial NOS (type 3 NOS) generates NO in the pulmonary vascular bed [9]. Upon generation, NO diffuses to adjacent cells [10]. NO’s half-life can be as low as 1.8 ms in the presence of mM concentrations of hemoglobin (Hb) [8, 11, 12], and therefore it is likely not the principal effector molecule for many NO bioactivities. NO can be rapidly oxidized to nitrate (NO_3_^-^) and nitrite (NO_2_^-^) [13], or taken by hemoglobin (Hb) to form nitrosyl-Hb (Hb:[Fe]NO) or S-nitrosothiol-Hb (SNO-Hb) [14].

NO administered via inhalation relaxes the pulmonary vasculature. Inhaled NO in critically ill neonates with pulmonary hypertension improves oxygenation [15, 16] and is currently approved by the US Food and Drug Administration [17, 18] for treatment of term and near-term neonates with hypoxic respiratory failure associated with clinical or echocardiographic evidence of pulmonary hypertension. Other studies have suggested that inhaled NO could be used to treat a wide spectrum of cardiopulmonary conditions, including acute respiratory distress syndrome (ARDS), chronic obstructive pulmonary disease (COPD), acute pulmonary embolism (PE), hypoxemic respiratory failure and pulmonary hypertension [19–23].

Inhaled, NO diffuses across the lung, reaching the smooth muscle cells of subjacent vessels where it causes selective pulmonary vasodilation [24, 25]. Besides local pulmonary effects, mounting evidence supports that inhaled NO exerts a variety of systemic effects. A number of studies have demonstrated that inhaled NO can affect multiple organs [26–35]. Indeed inhaled NO can be protective in brain injury as demonstrated by the hypoxia-ischemia or traumatic brain injury models [30–35]. Given the efficient scavenging by hemoglobin, the systemic effects are unlikely caused by direct effects of the inhaled NO itself [5]. Nitrite, low molecular weight SNO (eg. S-Nitrosoglutathione (GSNO)) or protein bound SNO species, may be involved in transporting the NO signal to the systemic circulation [36]. In fact, during hypoxic conditions, nitrite can regenerate NO by nitrite reductases [37, 38] and SNO-Hb is bioactivated by transfering nitrosonium (NO^+^) to other thiols [14, 39].

Controversy persists on whether both nitrite in plasma and SNO-Hb in erythrocytes increase during NO inhalation [40]. In fact, there remains a need for a quantitative evaluation of the array of circulating NO metabolites generated by breathing NO and the fate of these metabolites after ceasing its administration. Furthermore, little is known whether inhaled NO affects the cutaneous microcirculation. We hypothesized that inhaled NO increases the plasma levels of nitrite and SNO-Hb which then modify the cutaneous microvascular response to vasoactive mediators that challenge the NO pathway.

## Materials and methods

### a) Study approval

The study was approved by the institutional review board of the Cleveland Clinic (IRB # 12–1328). All subjects provided written informed consent prior to inclusion in the study. We conducted this cross-sectional study between May 2015 and September 2015.

### b) Subject selection and environment

Volunteers were in excellent general health and underwent a detailed evaluation to rule out smoking or conditions (e.g. diabetes, hypercholesterolemia or hypertension) that could affect the NO metabolism or cutaneous microcirculation. Subjects did not eat or drink for 4 hours prior to testing. Procedures were performed in a private room where individuals were acclimatized for at least 30 minutes (room temperature: 72° F or 22° C).

### c) Nitric oxide inhalation

Nitric oxide (INOMAX, Mallinckrodt Pharmaceuticals, Hazelwood, MO, USA) was administered continuously for 15 minutes through a disposable nasal cannula at a dose of 40 ppm, carried by room air at a flow rate of 4 L/min, following manufacturer’s recommendations.

### d) Laboratory determinations

#### -Blood collection

We placed a temporary venous access in the dorsal aspect of the right hand. Blood was obtained at baseline (before NO inhalation), at 15 minutes of continuous NO administration and at 5, 15 and 30 minutes of stopping this gas supplementation. Blood was collected using heparin vacutainer tubes (BD Biosciences, NJ, USA) and immediately centrifuged for 3 minutes to separate plasma from red blood cells (RBC). Without any delay, both plasma and RBC samples were frozen using liquid nitrogen, to preserve the NO metabolites. Samples were stored at -80° Celsius until assayed. Samples for analysis were only thawed once and the remainder discarded. No detectable nitrite was observed in the heparin vacutainer tubes or in sample storage tubes. All precautions were taken to minimize nitrite loss during the freezing / thawing procedure [41]. In addition, we tested the stability of nitrite levels during the freezing / thawing procedure by measuring nitrite before freezing and after snap freezing and thawing from a single individual and noted only a small variation in the nitrite concentration (fresh sample: 100.1 +2.6 nM and thawed sample: 101.4 +/- 4.8 nM (S1 Table)).

#### -Nitrite and nitrate determinations in plasma

Samples were deproteinated using methanol precipitation. Immediately after thawing the plasma samples, two volumes of methanol were added, and samples kept at -20° Celsius for at least 15 minutes to allow protein precipitation. Samples were then spun down to remove the precipitated proteins. The supernatant was then used for subsequent analyses. Nitrite was measured using ozone-based chemiluminescence with the triiodide method and Sievers NO analyzer (GE Analytical Instruments, Boulder, CO, USA) [42–44]. Briefly, the triiodide reagent was made using 1g of KCI and 0.65 g of iodine dissolved in 20 ml of distilled water, with the addition of 70 ml of acetic acid. A total of 6 ml of this solution was used in the reaction chamber for nitrite conversion into NO. A standard curve was generated using up to 500 nM of nitrite. We used 200ul aliquots of the sample to inject into the reaction chamber. Since the volume of the reaction chamber would alter over time, we limited the number of injections but kept the samples from each subject together. At the beginning with each fresh reagent in the reaction chamber, we ran a standard and one after the last sample. The levels of these standards were similar and so suggested the reagent was still sufficiently active. A similar method was used for detection of nitrate, with the only difference that the reagent was a saturated solution of vanadium (III) chloride (0.6 g in 100 ml of 1N HCl), heated to 94° C [45, 46]. A standard curve was generated using nitrate up to 25 uM.

#### -SNO-Hb and Hb:[Fe]NO measurement in RBCs

RBC samples were assayed for total, Fe-bound and thiol-bound NO (SNO) content by photolysis/chemiluminescence, as described previously [47, 48]. Briefly, samples were thawed (2 min, 37C), fully lysed (addition of ddH_2_O/vortex), and centrifuged (25,000 g, 5 min, 4C) to remove cell debris and membrane. Supernatant was run through a Sephadex G25 spin column, following which [Hb] was measured [49]. To selectively remove thiol bound NO (SNO), samples were incubated ± HgCl_2_ (6 fold molar excess over [Hb thiol]; tetramer basis)[50, 51]. Following this minimal processing, samples were injected into an HPLC pump (Analytical Scientific Instruments, Richmond, CA) purged with degassed, deionized water as the mobile phase, and carried (1 ml/min) to a custom photolysis system (Technosoft, Morrisville, NC, USA) comprising a quartz coil looped around a mercury arc lamp (Hanovia, Inc., Newark, NJ). This photolytically liberates bound NO, that is then subsequently carried in an inert gas stream (helium), to a high-resolution chemiluminescence NO analyzer (TEA 810, Ellutia, Charleston, SC, USA). Aqueous phase and higher oxides of nitrogen were removed in a series of cold traps interposed between the photolysis and analyzer units. A GSNO standard curve was performed each day with analysis of sample area under curve (Clarity, Madison, CT). Unknown sample areas were read off the standard curve, to determine NO content. Total NO content was derived from vehicle incubated sample. Fe-NO was obtained from the sample incubated with HgCl_2_. SNO-Hb was calculated from the difference between these two signals. Measurements of RBC NO are presented as a molar ratio of NO to Hb. Determinations were done in duplicate and an average reported [47, 48].

#### -Bicarbonate determination

Bicarbonate was measured using a Bicarbonate reagent kit on a Cobas C501 analyzer (Roche Diagnostics, Indianapolis, IN). Briefly, phosphoenolpyruvate in the presence of phosphoenolpyruvate carboxylase to produces oxaloacetate and phosphate. The oxaloacetate produced is coupled with NADH in the presence of malate dehydrogenase to produce malate and NAD. The consumption of NADH is measured 320 nm to 400 nm and the bicarbonate concentration determined.

#### -Cutaneous microvascular studies

Subjects were tested in a sitting position with the forearm at the level of the heart. We measured the subjects’ heart rate, blood pressure, and pulse oxygen saturation. We carefully prepped the skin of the anterior aspect of the forearm, 5 cm distal to the antecubital fossa and away from visible veins or skin abrasion [52]. Great care was taken to create similar experimental conditions to ensure regional and temporal reproducibility.

### a) Skin microvascular flow

We estimated the skin microvascular flow during the last minute of NO inhalation and for 5 minutes after its discontinuation, using Laser Doppler flowmetry [53] with the PeriFlux system 5000 (Perimed, Järfälla, Sweden) and an integrating probe (PF 413) [54]. We continuously recorded data from the instrument for off-line review, using the PeriSoft for Windows software. Flow measurements are expressed as arbitrary perfusion units (PU) averaged over 30 seconds of recording. Probes were calibrated using a motility standard consisting of a colloidal suspension of polystyrene particles (PF 1000). Laser Doppler flowmetry assesses the skin capillary perfusion (depth of skin penetration of about 1mm) by measuring the Doppler shift induced by the laser light scattering of moving red blood cells. Perfusion is defined as the concentration of red blood cells times their average velocity [55, 56].

### b) Iontophoresis of acetylcholine and sodium nitroprusside

Iontophoresis was performed using the thermostatic probe PF 481, the periIont micropharmacology system, the drug delivery electrodes PF 383 and the dispersive electrodes PF 384 (Perimed, Järfälla, Sweden) [52]. At different skin sites, we placed 180 μL of either acetylcholine 1% (Sigma-Aldrich, St Louis, MO, USA) or sodium nitroprusside 1% (Marathon Pharmaceuticals, LLC, Northbrook, IL, USA) in the sponge of the drug-delivery electrode. We iontophoresed these agents at 40 uA for 5 minutes. We used a positive polarity for acetylcholine and negative polarity for sodium nitroprusside. Both tests were performed before and during NO administration (at minutes 5 and 9 for acetylcholine and sodium nitroprusside, respectively). Current-induced vasodilation was prevented by limiting the current density to < 0.01 mA/cm^2^ [57]. During each test, we measured changes in PU and monitored skin resistance and temperature. Skin resistance was determined by the periIont micropharmacology system and recorded before and during the iontophoresis of medications, since variations in the skin resistance may affect the current flow and the transport of vasoactive mediators.

We believe that the cutaneous iontophoresis of vasoactive agents has not affected the levels of NO metabolites, given its local effect, low dose and the fact that blood was obtained in the opposite arm. Additionally, we have previously shown that using our methodology levels of iontophoresed agents are not detectable in blood [52].

### Statistics

Continuous data are presented as median (interquartile range (IQR)). Categorical data are summarized as discrete values and percentages (n (%)). Results of the iontophoresis tests are expressed as peak PUs or percentage of variation from baseline. Independent continuous samples were compared using Wilcoxon-Mann-Whitney test. Paired continuous samples were tested with Wilcoxon signed-rank test and Bonferroni correction. We tested repeated measurements with the nonparametric Friedman test. Relationships between normally distributed variables were assessed using the Spearman rank correlation coefficient. All *p* values are two-tailed and a value of < 0.05 was considered significant. The statistical analyses were performed using the statistical package IBM SPSS, version 20 (IBM; Armonk, New York) and MedCalc, version 14.12.0 (Ostend, Belgium).

## Results

### a) Nitric oxide metabolites

**Fig 1:**
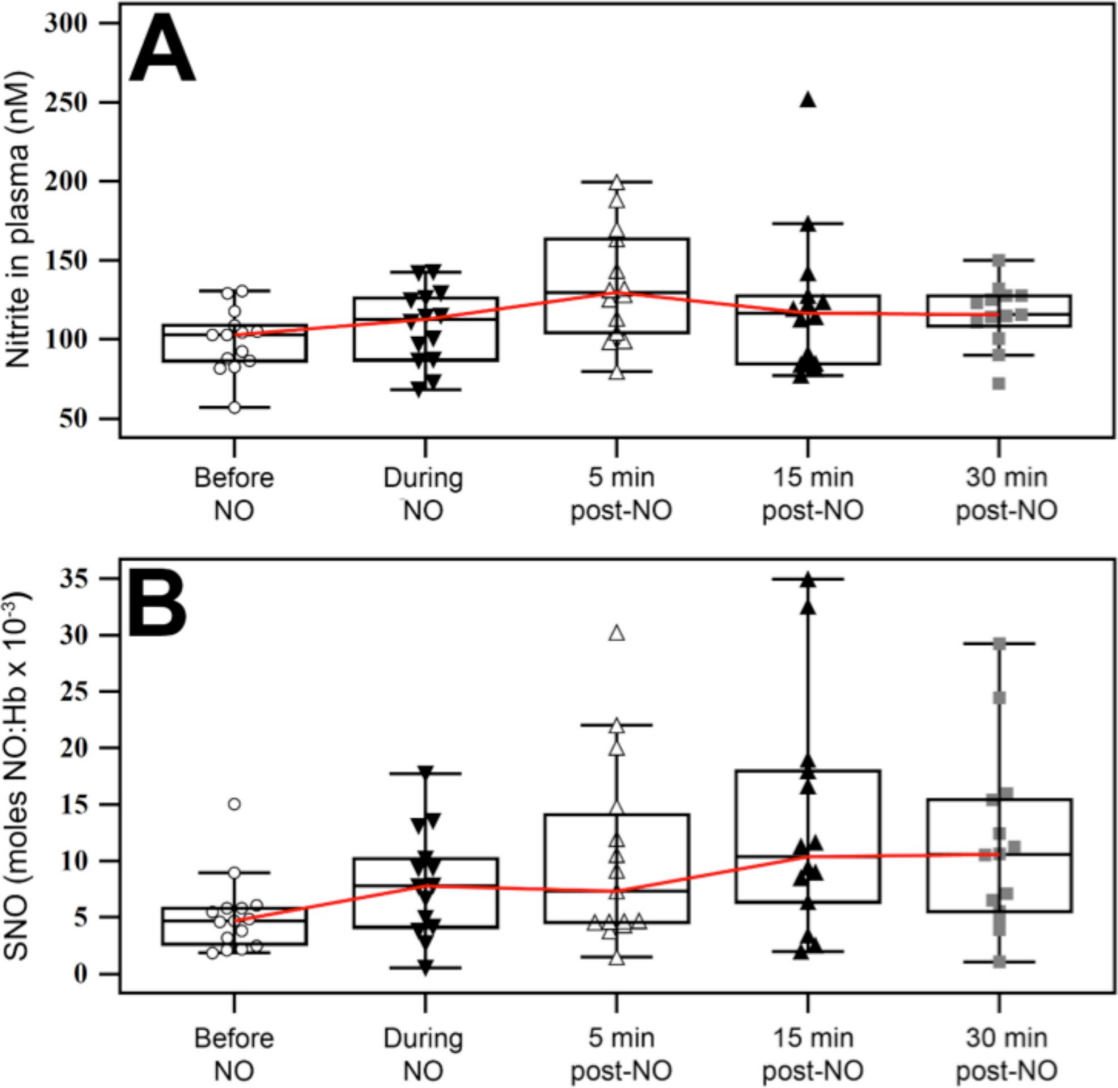
Box plots of plasma nitrite and RBC SNO-Hb before, during and after NO inhalation. The red line connects the median values at the different time points.

We included 15 subjects with a median (IQR) age of 37 (31–47) years, of whom 13 (87%) were females. We tested nitrite, nitrate, SNO-Hb and Hb:[Fe]NO before, during and after NO administration (Table 1 and Fig 1). The basal levels of nitrite and SNO-Hb are consistent to those found by previous investigators [48, 58–61]. The Friedman test showed significant differences among the various time points in nitrite, total RBC NO, Hb:[Fe]NO and SNO-Hb (Table 1). We observed a significant increase in nitrite (median (IQR) difference of 7 (-2 –+19) %, p = 0.048), total RBC NO (29 (-12 –+111) %, p = 0.02) and SNO-Hb (median (IQR) difference of 59 (-15 - +282) %, p = 0.02) during NO inhalation compared to baseline. Nitrite, total RBC NO and SNO-Hb levels were higher 5 minutes after the discontinuation of NO compared to baseline (28 (13–52) %, p = 0.009, 46 (3–96) %, p = 0.006, and 50 (-16 –+329) %, p = 0.03, respectively). Interestingly, the levels of nitrite peaked at 5 minutes; meanwhile, total RBC NO and SNO-Hb continued to increase during the 30 minutes after discontinuing NO.

**Table 1:** Determinations of NO metabolites in plasma and RBC.

		Baseline Median (IQR)	During NO inhalation* Median (IQR)	After 5 min of NO discontinuation Median (IQR)	After15 min of NO discontinuation Median (IQR)	After 30 min of NO discontinuation Median (IQR)	P (Friedman test)
**Plasma**	**n**	15	15	15	15	14^	
	**Nitrate** (uM)	25.4 (20.9–35.0)	26.6 (21.4–35.4)	27.3 (22.8–35.3)	27.4 (20.8–33.7)	26.9 (20.2–34.3)	0.07
	**Nitrite** (nM)	102.8 (86.3–117.8)	114.0 (87.0–129.0)	131.3 (104.3–169.5)	119.3 (84.8–141.8)	115.5 (105.8–127.5)	<0.001
**RBC**	**Total NO** (moles NO:Hb x 10^−3^)	13.2 (8.0–15.7)	15.3 (11.4–26.2)	15.2 (9.9–21.6)	18.2 (11.3–32.6)	18.7 (12.8–33.1)	0.005
	**NO[Fe]Hb** (moles NO:Hb x 10^−3^)	7.1 (3.0–11.8)	7.1 (3.7–14.6)	9.0 (2.8–12.2)	8.2 (4.7–9.8)	8.3 (5.4–19.0)	<0.001
	**SNO-Hb** (moles NO:Hb x 10^−3^)	4.7 (2.5–5.8)	7.8 (4.1–13.1)	7.3 (4.5–14.8)	10.3 (5.6–18.2)	10.6 (5.3–15.5)	0.005

*blood sample obtained at 15 minutes of continuous inhaled NO administration.

^ One patient had no blood sample 30 minutes after NO discontinuation.

**Abbreviations:** Hb:[Fe]NO: nitrosyl Hb, IQR interquartile range, NO: nitric oxide, O2: oxygen, SNO-Hb: S-nitrosothiol Hb.

### b) Bicarbonate determinations

In 11 subjects we measured bicarbonate in plasma before and 30 minutes after NO inhalation. The median (IQR) before NO inhalation and 30 minutes after discontinuation were 20.6 (19.8–23.4) and 20.5 (18.5–20.8), respectively (p = 0.04). The change in bicarbonate at 30 minutes was negatively associated with the change in SNO-Hb; however this association did not reach statistical significance.

### c) Forearm microvascular studies

The skin microvascular flow was not significantly different at baseline than during NO inhalation (Table 2). During NO inhalation, compared to before NO inhalation, the peak PU increased 62.8 (-218 - + 333.7) % points during the iontophoresis of acetylcholine and 95.8 (-123 - + 262.1) % points during the iontophoresis of sodium nitroprusside; changes that did not achieve statistical significance (Table 2).

**Table 2:** Forearm microvascular studies at baseline and during NO inhalation.

Variables	Baseline Median (IQR)	During NO inhalation Median (IQR)	P (Wilcoxon signed-rank test)
**Cutaneous PU**	13.6 (11.8–16.8)	14.6 (9.8–20.6)	0.28
**Acetylcholine iontophoresis**			
Baseline PU	5.6 (4.3–7.2)	4.5 (3.6–5.5)	0.33
Peak PU	49.6 (28.2–60.8)	47.4 (37.6–51.8)	0.87
Percentage change in PU	600 (420–965)	991 (363–1249)	0.57
Skin resistance	215 (175–279)	207 (165–261)	0.42
**Sodium nitroprusside iontophoresis**			
Baseline PU	4.1 (3.6–7.6)	4.2 (2.8–5.3)	0.31
Peak PU	17.1 (10.3–21.1)	11.6 (7.1–25.9)	0.96
Percentage change in PU	190 (127–407)	231 (107–424)	0.36
Skin resistance	241 (220–251)	222 (208–272)	0.49

Before NO inhalation, the peak PU and increase in PU during acetylcholine iontophoresis showed a negative association with the level of SNO-Hb (r: -0.57, p = 0.03 and r: -0.61, p = 0.03, respectively). Similarly, during NO administration, the peak PU and increase in PU during acetylcholine iontophoresis was negatively associated to the level of SNO-Hb under the same condition (r: -0.54, p = 0.04 and r: -0.51, p = 0.05, respectively). The change in peak PU during acetylcholine before and during NO inhalation was inversely related to the change in SNO-Hb (r: -0.57, p = 0.03). The levels of nitrite were not significantly associated with the acetylcholine iontophoresis response before or during NO administration.

## Discussion

**Fig 2:**
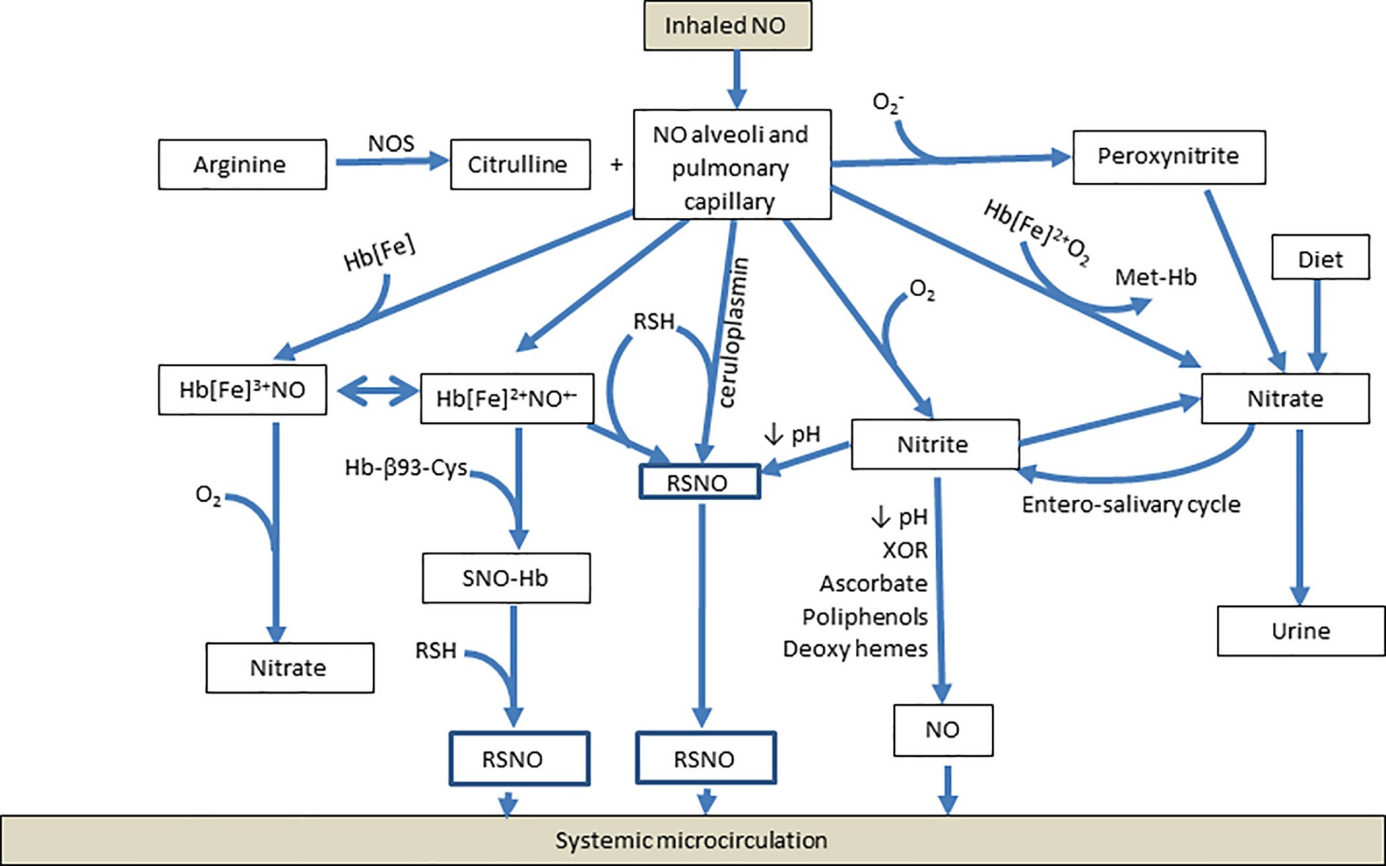
Mechanisms by which inhaled NO acts on the systemic microcirculation. Abbreviations: Hb: hemoglobin, Hb:[Fe]NO: nitrosyl Hb, Met-Hb: methemoglobin, NO: nitric oxide, NOS: nitric oxide synthase, O_2_: oxygen, ROS: reactive oxygen species, RSH: alkyl thiols, RSNO: S nitrosothiols, SNO-Hb: S-nitrosothiol Hb, XOR: xanthine oxidoreductase.

It is postulated that many of the systemic responses to inhaled NO are derived from its metabolites such as nitrite, S-nitrosothiols and possibly dinitrosyl iron complexes (DNICs). Both nitrite and S-nitrosothiols can be converted back to NO to deliver this molecule to sites remote to its generation. Nitrite can be reduced to NO by nitrite reductases and by low pH in ischemic tissues, whereas SNO-Hb can transfer NO^+^ under low oxygen concentrations in the peripheral circulation [14] (Fig 2). In the present study, we investigated how these metabolites changed during and 30 minutes after discontinuing a 15 minute inhalation of NO. To the best of our knowledge, the kinetics of inhaled NO increasing levels of SNO-Hb, have not been measured. We noted that the inhalation of NO led to a significant increase in nitrite and SNO-Hb. Nitrite peaked at 5 minutes and SNO-Hb peaked beyond 15 minutes, of discontinuing the NO inhalation. In fact, nitrite levels rapidly declined after its peak (consistent with the suggested half-life of 30 minutes) [59, 62]; while SNO-Hb levels remained elevated for at least 30 minutes after NO discontinuation, theoretically storing the NO signal for a longer time and possibly increasing the minute ventilation.

Conventionally, most excess inhaled NO reacts with Fe^++^ Hb and oxygen to form methemoglobin and nitrate; the methemoglobin is then recycled by methemoglobin reductases [40, 63]. Strikingly, we did not notice a significant change in plasma nitrate during NO inhalation. Many factors can affect nitrate levels, including diet and microbiome [64]. Inhalation of NO at the concentration given would produce at best a small and likely non-significant change given the inherent noise of the assay used.

In mice, the inhalation of NO led to increases in nitrate and nitrite in plasma as well as SNO-Hb and Hb:[Fe]NO in erythrocytes [65], with a plateau achieved within 15 minutes of administering NO [65]. In patients undergoing liver transplantation [66], inhaled NO produced an increase in plasma nitrate and nitrite as well as erythrocyte Hb:[Fe]NO, but not SNO-Hb. In contrast, our study showed an increase in SNO-Hb during NO inhalation, and the discrepancy may be in part related to the difficulties in measuring SNO-Hb using iodine-based methods [67], NO dosing and length of NO administration.

Initial studies suggested that the effects of inhaled NO were confined to the lungs given its high affinity for the heme moiety of Hb [68]. Subsequent investigations, in animals, showed that inhaled NO inhibits platelet function [69], increases glomerular filtration rate[70], reduces neointimal formation in injured arteries [71], enhances coronary artery patency after thrombolysis [72], reduces myocardial infarction size [73], and maintains mesenteric blood flow in intestinal ischemia-reperfusion injury [74]. Meanwhile, in humans, inhaled NO was noted to reduce muscle inflammation during limb ischemia [75] and ischemia-reperfusion injury during liver transplantation [66]. Of concern, long-term follow-up of infants exposed to inhaled NO suggests an increased cancer risk later in childhood [27]. Nitric oxide itself can be mutagenic, but most authors argue that NO radical is not present in any relevant concentration in the presence of mM vascular concentrations of Hb. The exception may be in ischemic tissue, where nitrite could be converted to NO by protonation. Thus, the peripheral oncogenic effects of inhaled NO could result from delivery of nitrite to ischemic tissues [76]. The peripheral physiologic effects of inhaled NO are typically dose- and oxygen tension-dependent and can occur without systemic hemodynamic changes [26, 77].

Nitric oxide equivalents are transferred as NO^+^ from deoxyhemoglobin to low molecular weight thiols in erythrocytes [14, 78–81]. These low molecular weight S-nitrosothiols are hypoxia-mimetic, signaling Hb R to T conformational change [14, 78–81]. One of these hypoxia-mimetic effects is to increase minute ventilation [78, 82, 83]. To maintain pH, hyperventilation-induced respiratory alkalosis will normally result in decreased bicarbonate levels. A decrease in steady state bicarbonate of 0.1 to maintain steady-state pH, by Henderson-Hasselbalch, corresponds to a decrease in steady state pCO_2_ of 0.5 mmHg. By Dubois, this corresponds to a steady increase in minute ventilation of ~ 0.5 litres per minute at steady state pO_2_. Though this normal physiology is not observed in critically ill patients who are mechanically ventilated, we were able here, uniquely, to measure the decrease in bicarbonate associated with excess NO-hemoglobin loading in spontaneously breathing, healthy subjects.

The iontophoresis of acetylcholine and sodium nitroprusside test the microvascular endothelial-dependent and independent vasodilation, respectively [84]. Acetylcholine induces vasodilation via the endothelial production of NO and prostanoids, pathways that likely participate in enzymatic cross-talk [85]. Sodium nitroprusside reacts with tissue sulfhydryl groups to produce NO directly (NO donor) [85]. In our study, the inhalation of NO did not significantly affect the microvascular cutaneous perfusion or the response to the iontophoresis of acetylcholine or sodium nitroprusside. These findings could represent that the microvascular studies used were not sensitive and/or precise enough to detect the systemic microvascular effects of inhaled NO, and/or the normal tissue oxygen tension of skin tissue. Another explanation could be that inhaled NO elicits a more pronounced microvascular response in states of oxidative stress, hypoxic tissues or decreased NO production than in healthy controls [74]. In fact, Cannon III et al [40] noted that NO inhalation produced minimal effects on the forearm blood flow; however, inhaled NO reverted the reduced forearm blood flow caused by the blockade of NO synthesis with NG-monomethyl-L-arginine [40].

Prior investigations in healthy individuals showed that stimulation with acetylcholine augmented forearm blood flow and venous nitrite [64, 86]. Although we showed no significant changes in the microvascular studies during the inhalation of NO, we noted an inverse association between the levels of SNO-Hb and the response to acetylcholine iontophoresis both before and during NO administration. A higher RBC level of SNO-Hb may represent a higher NO state with less microvascular response to the iontophoresis of acetylcholine. Interestingly, the more pronounced the increase in SNO-Hb during NO inhalation the lower the change in response to acetylcholine iontophoresis between baseline and NO administration.

Note that under conditions of extreme oxidative stress in the lungs, i.e. acute respiratory distress syndrome, inhaled NO has adverse effects on renal function, whereas during heart surgery without lung injury, inhaled NO may benefit renal function [28, 29]. This difference suggests that airway chemistry is important to the metabolic fate and systemic effects of inhaled NO [14] (Fig 2). Consistent with this hypothesis, SNO-Hb levels continued to increase after the inhaled NO was discontinued. The duration of this increase was longer than a circulatory cycle, arguing against simple transfer of NO from Fe^++^ Hb to Hb-thiol as the principle reason for this steady post-dose increase [26]; and stoichiometrically, the increase in SNO-Hb occurred without any loss of RBC NO. These data almost certainly argue for a capacitor in the circuit: a reservoir of SNO in the airways that stores NO^+^ equivalents and transfers them to the blood. In fact, airway reduced thiol levels are in excess of 100 μM, and formation of a major SNO reservoir from endogenous thiols has previously been demonstrated in the distal airway during NO inhalation in humans and in pigs [87, 88].

Limitations of the current study include a) a relatively small number of healthy volunteers (n = 15) that might have prevented the identification of significant differences in microvascular studies or NO metabolites, and b) microvascular studies were not repeated after the discontinuation of NO when the levels of nitrite and SNO-Hb peaked. However, for the first time, we rigorously measured in healthy volunteers both nitrite and SNO-Hb at baseline, during and up to 30 minutes after the discontinuation of NO inhalation. Our data show that both nitrite in plasma and SNO-Hb in RBC increase during and immediately after NO inhalation and that these metabolites may affect studies that test the NO pathway in the systemic microvasculature.

## Conclusions

For the first time we measured the kinetics of nitrite and SNO-Hb during NO inhalation and after its discontinuation. Interestingly, the kinetics of these NO metabolites are not identical. Nitrite increases first, followed by a more sustained increase in Hb-SNO, likely reflecting the capacitor-like reservoir of SNO in the lung. Nitrite, Hb-SNO and possibly DNICs could be a mobile reservoir of NO with implications on the systemic microvasculature.

## Supporting information

S1 Table Effect of freezing/thawing on nitrite measurements. This is a table with the measurements of nitrite from samples that were fresh or frozen/thawed from a single individual. This demonstrated that the nitrite levels were not affected by freezing and thawing under the condition we used.
(XLSX)

S2 Table Measurement of SNO, Nitrite, and Nitrate. This excel sheet contains the data for the measurements of SNO, nitrite and nitrate for the different samples. It also contains the standard curves along with the associated R^2^ values and the calculated values of the samples.
(XLSX)

S3 Table Collated data for NOx metabolites, CO_2_ and iontophoresis related measurements. In addition to the different NOx metabolites, CO_2_ and iontophoresis results it also includes data related to age, gender and BMI status of the subjects.
(XLSX)
